# Goal Orientation and the Presence of Competitors Influence Cycling Performance

**DOI:** 10.3389/fpsyg.2018.01212

**Published:** 2018-07-23

**Authors:** Andrew W. Hibbert, François Billaut, Matthew C. Varley, Remco C. J. Polman

**Affiliations:** ^1^Institute for Health and Sport, Victoria University, Melbourne, VIC, Australia; ^2^College of Sport and Exercise Science, Victoria University, Melbourne, VIC, Australia; ^3^Department of Kinesiology, Laval University, Quebec, QC, Canada; ^4^School of Exercise and Nutrition Sciences, Queensland University of Technology, Brisbane, QLD, Australia

**Keywords:** pacing, time-trial, motivation, ego, task

## Abstract

**Introduction:** The aim of this study was to investigate time-trial (TT) performance in the presence of one competitor and in a group with competitors of various abilities.

**Methods:** In a randomized order, 24 participants performed a 5-km cycling TT individually (IND), with one similarly matched participant (1v1), and in a group of four participants (GRP). For the GRP session, two pairs of matched participants from the 1v1 session were used. Pairs were selected so that TT duration was considered either inferior (INF) or superior (SUP) compared to the other pair of participants.

**Results:** Overall, TT duration (*P* = 0.86, $\eta_{p}^{2}$ < 0.01) was not different between conditions, while heart rate (HR) was significantly greater in GRP compared to IND (*P* < 0.01, $\eta_{p}^{2}$ = 0.16). For INF, a large effect size for both mean power (*P* = 0.07, $\eta_{p}^{2}$ = 0.15) and HR (*P* = 0.05, $\eta_{p}^{2}$ = 0.16), indicates greatest effort in GRP. Pacing behavior was affected by competition but similar in 1v1 and GRP for SUP, while large effect sizes indicate an increased power output in the initial 750-m for INF in GRP. Additionally, for INF, there was a significant correlation with ego orientation for an increase in TT duration between the GRP session and both the IND (*r* = 0.43, *P* = 0.04) and 1v1 (*r* = 0.54, *P* = 0.01) sessions.

**Conclusion:** For INF participants, intensity was increased when competing in GRP. Yet, the presence of the SUP competitors resulted in lesser performance improvements for ego oriented INF participants. These findings demonstrate that consideration should be given to the ability of competitors in a group setting to provide adequate motivation.

## Introduction

During exercise, an individual will regulate intensity to achieve personal goals and optimal performance while limiting the possibility of early exhaustion (St Clair Gibson et al., 2006). This process of pacing involves the consideration of many circumstantial factors to set and regulate an appropriate exercise intensity (Edwards and Polman, 2013; Smits et al., 2014). In competitive exercise situations, in addition to managing neuromuscular fatigue, pacing and overall performance are likely to be influenced by the presence or perception of a competitor (Triplett, 1898; Renfree et al., 2014; Hettinga et al., 2017).

In comparison to exercising alone, cycling time-trial (TT) performance is improved when exercising in the presence of a competitor (Triplett, 1898) and a virtual avatar competitor that represents a previous performance (Corbett et al., 2012; Stone et al., 2012). In addition, deceptively faster avatars as a motivational stimulus have resulted in increased performances (Stone et al., 2012; Williams et al., 2015a,b; Jones et al., 2016a,b). Furthermore, unsustainable behavior (i.e., a fast start) of avatar competitors has enticed participants to change their pacing strategy (Konings et al., 2016). Taken together, these results demonstrate that perception of competitors is an important consideration for pacing decisions, and this provides a stimulus that motivates for a greater performance. Yet, within these studies, by competing against a virtual avatar, the psychological dynamics such as social facilitation that actual competition provides, are not present (Bond and Titus, 1983; Snyder et al., 2012). In fact, the presence of real competitors increases arousal and attentional processes, resulting in an increased exercise intensity in comparison to a virtual stimulus (Ravaja et al., 2006; Snyder et al., 2012). Previously, within pacing studies, the presence of competition has been imitated with a concealed dummy rider to deceive the participant that an avatar was an actual competitor (Corbett et al., 2012). Yet, the use of actual competition has been limited to a small number of investigations with equivocal results (Bath et al., 2012; Tomazini et al., 2015). A competitor mimicking the movement of the investigated runner did not improve running TT performance, as this was deemed an unsuitable motivational stimulus (Bath et al., 2012). In a more realistic competitive setting, running as a group of four to five matched participants resulted in a reduction of TT duration, with improvements attributable to a greater starting speed (Tomazini et al., 2015). However, the use of participants with similar performances limits the comparison between differing ability participants that would likely occur in a group competition setting. Additionally, from this study, it is not apparent if this group competition is more beneficial to performance than the competition provided by a similarly matched competitor. Nevertheless, for pacing considerations, actual competition creates athlete interactions allowing for opportunities to act, or to respond to the actions of competitors (Smits et al., 2014). In addition, the presence of group competition creates multiple athlete interactions as opposed to one competitor. However, a comparison between one competitor and many competitors in an actual competition setting has not yet been investigated.

Social facilitation theory provides an explanation of why performance alone or in the presence of others might differ (Bond and Titus, 1983; Strauss, 2002; Snyder et al., 2012). Furthermore, in a group setting, multiple inter-individual differences in perceptions of competence and ability will create different approaches to a task, based upon motivational orientation and personal goals (Nicholls, 1984; Smits et al., 2014). Goal orientation theory refers to how individuals estimate their levels of ability and effort within a task (Duda, 1992). Based on the two perspectives of goal orientation theory (ego and task), individuals are likely to approach exercise tasks differently. An ego orientated individual will emphasize winning and might demonstrate different behavior to a task orientated individual who emphasizes learning and improvement (Duda et al., 1991; Chi and Duda, 1995). Additionally, motivational orientation has been suggested to influence competitive behavior. Intrinsic motivation will be a key driver for performance improvements, yet competition may influence perceptions of competence that may reduce intrinsic motivation (Ryan and Deci, 2017). Consequently, in examining the difference between competitive settings, it would be of interest to investigate goal perspectives and motivational orientation to clarify if responses to competition are similar or whether different ability opponents influence behavior and decisions of the competing athlete.

The primary aim of this study was to investigate how pacing and performance are influenced when exercising in the presence of one competitor or multiple competitors. The secondary aim was to investigate the influence of goal orientation on the magnitude of performance change by manipulating the ability of competitors in a group setting. It was hypothesized that performance in a group setting would be improved compared to a session with one competitor, and that ego orientated participants would have greater performance improvements when exposed to competition, compared to task orientated participants. No predictions were made for the role of motivational orientation. To create a competitive environment, participants cycled on a stationary bike adjacent to competitors, with performance projected onto a monitor.

## Materials and Methods

### Experimental Overview

Participants reported to the laboratory on eight occasions, which included five preliminary and three experimental sessions. To assess cardiorespiratory fitness, the first two preliminary sessions involved two incremental exercise tests to determine peak oxygen uptake (VO_2peak_), the first being a familiarization (FAM; see procedure below). As previous cycling experience varied between the participants, three FAMs to the 5-km cycling TT were performed to develop a reproducible pacing strategy and performance (Hibbert et al., 2017). For experimental testing, on three different days separated by a minimum of 48 h, participants performed three 5-km cycling TT’s in a randomized order: An individual 5-km TT (IND), a 5-km TT performed with another matched participant (1v1), and a 5-km TT performed in a group setting with four participants (GRP) (see procedure below).

### Participants

In total 24 (12 females and 12 males) recreationally active participants (**Table 1**) volunteered to take part in this study and provided written informed consent in accordance with the Declaration of Helsinki. Victoria University’s Human Research Ethics Committee provided ethical approval for this study and all procedures were conducted in accordance with the recommendations of the National Statement on Ethical Conduct in Human Research as described by the National Health and Medical Research Council (NHMRC) of Australia. Prior to commencing the study, all participants were screened for suitability to the exercise protocol and risk factors using a medical questionnaire. Participants were asked to refrain from any physical activity causing severe fatigue in the 36 h prior as well as any caffeine intake 2 h prior to testing.

**Table 1:** Participant anthropometric data.

Measure	INF	SUP	Total	*P*-value
	*n* = 12	*n* = 12	*n* = 24	
Age (years)	24.58 ± 4.98	26.58 ± 4.10	25.58 ± 4.58	*P* = 0.30
Height (cm)	169.42 ± 6.49	176.25 ± 11.96	172.83 ± 10.04	*P* = 0.10
Body mass (kg)	69.96 ± 12.42	73.88 ± 15.75	71.92 ± 14.02	*P* = 0.51
PPO (W)	277.83 ± 54.13	316.58 ± 67.63	297.21 ± 63.09	*P* = 0.14
PPO (W/kg)	4.00 ± 0.65	4.32 ± 0.66	4.16 ± 0.66	*P* = 0.24
VO_2peak_ (ml.min.kg^-1^)	44.24 ± 7.88	47.92 ± 8.68	46.08 ± 8.32	*P* = 0.22
VO_2peak_ (L.min^-1^)	3.10 ± 0.77	3.54 ± 0.93	3.32 ± 0.87	*P* = 0.29
Ego	2.70 ± 0.99	2.73 ± 0.98	2.72 ± 0.96	*P* = 0.93
Task	4.40 ± 0.50	4.52 ± 0.41	4.46 ± 0.45	*P* = 0.54

*Data presented as mean ± SD. Each group *n =* 12, consisting of *n =* 6 females and *n =* 6 males. PPO, peak power output; VO_*2peak*_, peak oxygen consumption; Ego, the mean of ego responses from the GOEM; Task, the mean of task responses from the GOEM; GOEM, goal orientations of exercise measure; INF, inferior; SUP, superior.*

### Participant Characterization

#### VO_2_ Assessment

VO_2peak_ was assessed using a 30 Watts/min ramp maximal incremental test after a 3-min baseline period cycling at 0 Watts (Vanhatalo et al., 2010). Expired gas was collected and analyzed every 15-s [S-3A/I (O_2_) and CD-3A (CO_2_), AEI Technologies Inc., Pittsburgh, PA, United States], with gas and flow calibrations performed prior to each test. The test concluded when the participant could no longer maintain a cadence above 60 rpm or volitional fatigue was achieved, with the participants encouraged throughout the final stages of the test. VO_2peak_ was calculated as the highest 30-s mean VO_2_ and peak power was defined as the highest power at test conclusion. As cycling experience between the participants varied, two incremental tests were conducted to ensure familiarity with the protocol.

#### Participant Matching

The best performance (TT duration) from the three FAM TT’s was used to match participants for the competition sessions. Participants were matched based on sex to remove any possible physiological and perceptual influences of sex on the competition. Initially, participants were matched to a similar participant for the 1v1 session. These participants had a TT time that was overall between 16.41 ± 16.97-s of each other’s best FAM TT duration. For the GRP session, two pairs of matched participants from the 1v1 session were used to make a group of four participants. In the GRP session, pairs of participants were selected so that one pair had a 5-km TT duration that was either considerably slower (between 110 and 120% of TT duration), or considerably faster (between 80 and 90% of TT duration) than the other pair of participants. For this session, the slower participants were categorized as inferior (INF), while the faster participants were categorized as superior (SUP). Overall for the GRP session matching, the SUP participants were 58.96 ± 22.09-s faster than the INF participants.

#### Goal Orientation

After the first FAM TT, participants completed a 10 question Goal Orientations in Exercise Measure (GOEM) to assess individual differences in goal perspectives in exercise settings (Petherick and Markland, 2008). The GOEM was used to assess ego and task orientation, with the measure consisting of two subscales, with five questions measuring ego and five questions measuring task orientation. Participants responded on a 5-point Likert scale ranging from 1 (*strongly disagree*) to 5 (*strongly agree*). Orientation was measured as the mean of responses to the five subscale questions. The GOEM has shown to have strong psychometric properties and reliability (Petherick and Markland, 2008).

### Time-Trials

All exercise was conducted on Velotron Pro cycle ergometers (RacerMate Inc., Seattle, WA, United States), that had been fitted with a scientific SRM power meter (Schoberer Rad Meßtechnik, Jülich, Germany). A calibration of the power meter was conducted before each test. Power output and heart rate (HR) were collected with a wireless Power control 7 unit and later downloaded using the SRM training system (Schoberer Rad Meßtechnik, Jülich, Germany). All TT protocols were controlled using the Velotron Interactive 3D software (Version 1.0, RacerMate Inc., Seattle, WA, United States) where the performance of the participant was projected onto a monitor. To increase visibility, the computer monitor was projected onto a 42″ monitor, placed in front of the participant. All FAM and IND sessions were conducted with the cycle ergometer positioned central to the monitor behind the participant’s avatar. For the 1v1 session, the cycle ergometer was again positioned behind the avatar, so that participants were approximately 1 m apart. For the GRP session, as the 3D software only allows for two participants, two separate computers and monitors were used so that two participants were displayed on one monitor and the other two were on another. In this session, participants were split from their 1v1 participant so that on each monitor there was an INF and a SUP participant.

Within the first FAM session, participants set the ergometer to their own specifications with values recorded and replicated for subsequent sessions. Upon arrival to the laboratory for experimental trials, participants completed a warm-up consisting of 5-min of cycling at 75 Watts. To overcome flywheel inertia, participants were instructed to obtain a self-selected comfortable cadence immediately prior to beginning the TT, with the TT commencing after a verbal 3-s countdown from the researcher. Participants could change gear and cadence throughout the TT as desired with the instruction to finish the required distance “as quickly as possible.” For the competition sessions, there was no instruction or incentive for the participant to beat their competitors. Instead, the participants were instructed to finish the required distance “as quickly as possible” in the presence of other competitors (Williams et al., 2015b). Participants were blinded from all information except for the distance covered, yet in the 1v1 and GRP sessions, participants could also see the distance covered by competitors as well as visual proximity via the computer avatar. Upon TT completion, participants were instructed to remain on the ergometer until all participants had completed the required distance.

### Motivational Orientation and Perceptual Scores

After completion of each TT, a 17-item version of the intrinsic motivation inventory (IMI) (McAuley et al., 1989) was used to assess interest/enjoyment, perceived competence, and pressure/tension during that trial. Participants responded on a 7-point Likert scale ranging from 1 (*Not true at all*) to 5 (*Very true*). The IMI has been shown to have strong factor structure and reliability (McAuley et al., 1989). During each TT, at every kilometer, participants were asked to rate perceived exertion (RPE) (Borg, 1970) and affect (Hardy and Rejeski, 1989). Scales were placed adjacent to the monitor and in full view during the TT. Prior to commencing the study all scales were explained to participants.

### Statistical Analysis

Experimental TTs are defined as a TT conducted individually (IND), with a similarly matched participant (1v1) and a session where one pair of slower participants completes a TT with a pair of faster participants (GRP). Slower participants for the GRP session are defined as inferior (INF) and faster participants are defined as superior (SUP). All data was analyzed using SPSS (version 22, SPSS Inc., Chicago, IL, United States) with all data reported as mean ± SD. Statistical significance levels for all tests was set at *P* < 0.05. Tests for homogeneity of variances were performed to ensure normality of the cohort for dependent variables. When homogeneity of variances was violated, Welch *F*-ratio is reported for analysis of variance (ANOVA). When normality assumptions were violated for Pearson correlation coefficient (*r*), Spearman’s rho (*r_s_*) was calculated. In the instance of a significant ANOVA, *post hoc* Sidak comparisons were conducted. Effect sizes for ANOVAs are reported as partial eta squared ($\eta_{p}^{2}$) with a small effect at 0.01–0.059, a medium effect at 0.06–0.139 and a large effect >0.14. Effect sizes for correlations are reported as Pearson’s *r* with a small effect at 0.10–0.29, a medium effect at 0.30–0.49 and a large effect >0.5. Effect sizes for *t*-tests are reported as Cohen’s *d* with a small effect at 0.2–0.49, a medium effect at 0.5–0.79 and a large effect >0.8 (Cohen, 1988).

#### Preparation for Data Analysis

Given the inter-participant differences in TT power output, power has been reported as a percentage of the individual’s PPO obtained from the maximal incremental test (i.e., % of PPO). As the IND and 1v1 sessions followed the same conditions for all participants, for the analysis of variables between sessions, group classification was ignored so that in each session *n* = 24. For correlation analysis, due to the inter-participant differences created by the study design (i.e., INF and SUP participants), overall TT duration has been calculated as differences between sessions. Changes in performance are defined as the difference between IND and 1v1 sessions (IND-1v1), the difference between IND and GRP sessions (IND-GRP) and the difference between 1v1 and GRP sessions (1v1-GRP).

#### Analysis of Overall Performance

To examine any differences between participant characteristics, an independent sample *t-*test was conducted on group (INF and SUP) anthropometric and goal orientation variables. To explore whether there was an influence of competition on TT performance measures, a one-way ANOVA (three conditions) on TT duration, mean power and mean HR was conducted. To investigate differences created by the competitive stimulus (i.e., the difference in ability between INF and SUP), a one-way ANOVA for both INF and SUP groups was conducted. Based on the hypothesis of the GRP competition (i.e., SUP being a competitive stimulus for INF participants), an independent samples *t*-test was conducted between the INF and SUP groups for the difference in TT duration between the 1v1 and GRP sessions.

#### Analysis of Goal Orientation

To investigate the influence of participant goal orientation (ego and task) on overall performance, Pearson product moment correlation coefficients (*r*) were calculated for ego and task scores from the GOEM and changes in TT duration between sessions. Pearson correlations were conducted on the whole group and the INF and SUP groups separately.

#### Analysis of Motivational Orientation and Perceptual Scores

To analyze if the competitive settings influenced IMI responses, exertion and affect between trials, a one-way ANOVA was conducted on mean IMI responses (*n* = 24) and for RPE and FS at each kilometer. To investigate the variation created by differences in competitive stimulus (i.e., the difference in ability between INF and SUP), we conducted a one-way ANOVA for both INF and SUP groups for IMI responses, and for RPE and FS at each kilometer. To compare the difference in perceptual scores between INF and SUP within the GRP session, we conducted an independent samples *t*-test for IMI responses and for RPE and FS at each kilometer.

#### Analysis of Pacing Profiles

To compare pacing profiles, power output data was averaged over 250-m intervals, with one-way ANOVA’s conducted for the mean of all participants (*n =* 24) at each 250-m interval. To compare power output changes within groups between trials (*n =* 3), one-way ANOVAs for each 250-m interval were conducted for the INF and SUP groups.

To investigate the influence of participant goal orientation (ego and task) on the change in pacing behavior, *a posteriori* analysis of Pearson product moment correlation coefficients (*r*) were calculated for ego and task scores from the GOEM and changes in power output at 250-m intervals between sessions. Pearson correlations were conducted on the group and the INF and SUP groups separately.

## Results

### Participant Matching

There was no significant difference between the INF and SUP groups for any anthropometric variable (**Table 1**). Within the 1v1 session, the mean difference between participants was 19.64 ± 17.84-s. Within the GRP session, the mean difference between the pairs of participants was 17.33 ± 14.60-s, whilst the difference between the SUP and INF participants was 44.83 ± 18.53-s.

### Analysis of Overall Performance

**FIGURE 1:**
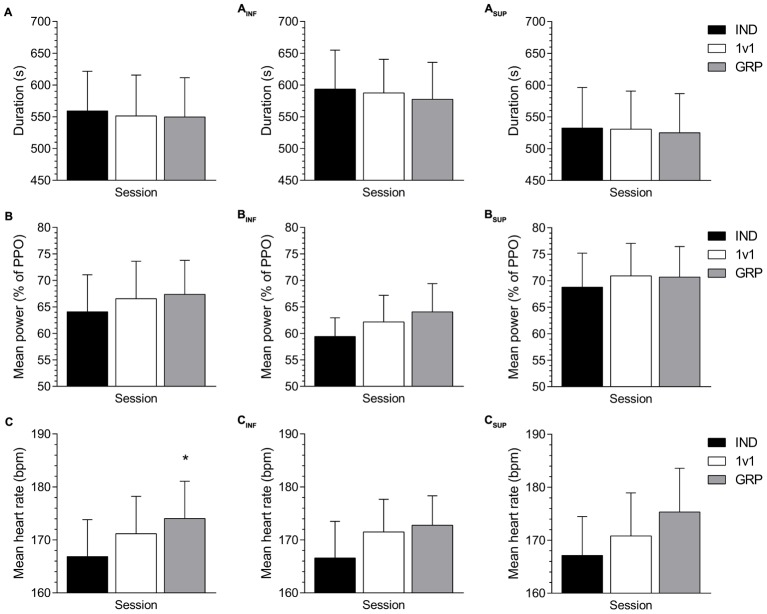
TT overall performance measures. Mean changes in TT duration **(A)**, mean power **(B)**, and mean HR **(C)** between IND (black), 1v1 (white), and GRP (gray) sessions. Mean changes for INF participants in TT duration **(A_INF_)**, mean power **(B_INF_),** and HR **(C_INF_)**. Mean changes for SUP participants in TT duration **(A_SUP_)**, mean power **(B_SUP_),** and HR **(C_SUP_)**. INF, inferior; SUP, superior; IND, individual TT; 1v1, two matched participants TT; GRP, TT with four participants (two INF and two SUP participants). ^∗^Significant difference to IND.

**FIGURE 2:**
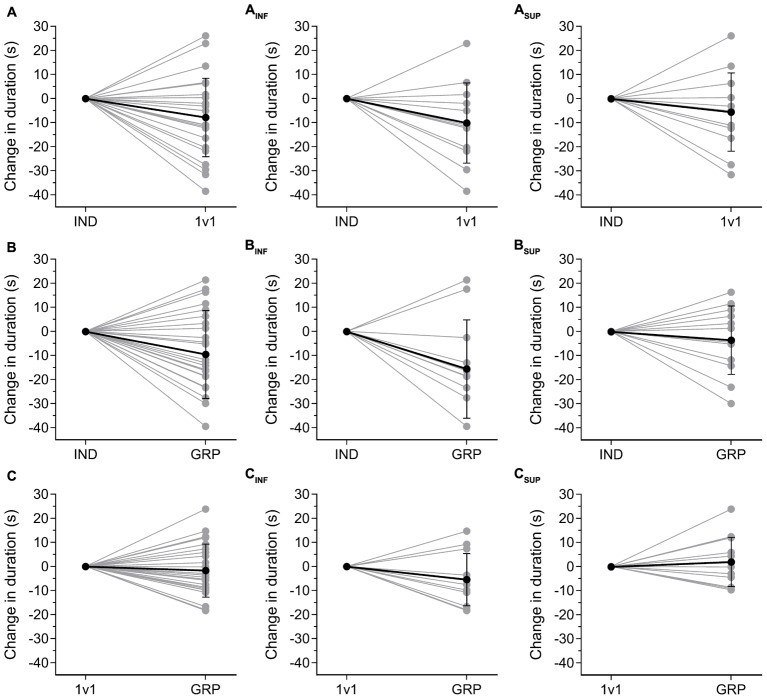
Individual changes in TT duration between sessions. **(A–C)** Change in TT duration for all participants (*n* = 24) between IND and 1v1 **(A)**, IND–GRP **(B)**, and 1v1–GRP **(C)**. **(A_INF_–C_INF_)** Changes in TT duration for INF participants (*n* = 12) between IND and 1v1 **(A_INF_)**, IND–GRP **(B_INF_)** and 1v1–GRP **(C_INF_)**. **(A_SUP_–C_SUP_)** Changes in TT duration for SUP participants (*n* = 12) between IND and 1v1 **(A_SUP_)**, IND–GRP **(B_SUP_)** and 1v1–GRP **(C_SUP_)**. INF, inferior; SUP, superior; IND, individual TT; 1v1, two matched participants TT; GRP, TT with four participants (two INF and two SUP participants).

Overall, there was no significant effect for TT duration (*P* = 0.86, $\eta_{p}^{2}$ < 0.01) or mean power (*P* = 0.23, $\eta_{p}^{2}$ = 0.04) between conditions (**Figures 1A,B, 2A–C**). However, mean HR was significantly greater in GRP compared to the IND session (*P* < 0.01, $\eta_{p}^{2}$ = 0.16) (**Figure 1C**).

For the INF group, there was no significant difference for TT duration (*P* = 0.79, $\eta_{p}^{2}$ = 0.02), mean power (*P* = 0.07, $\eta_{p}^{2}$ = 0.15) and HR (*P* = 0.05, $\eta_{p}^{2}$ = 0.16). There were, however, large effect sizes for mean power and HR. Visualization of data suggests there was increased mean power (**Figure 1B_INF_**) and HR (**Figure 1C_INF_**) in the GRP session. For the SUP group, there was no significant difference in TT duration (*P* = 0.98, $\eta_{p}^{2}$ < 0.01), mean power (*P* = 0.64, $\eta_{p}^{2}$ = 0.03) and HR (*P* = 0.05, $\eta_{p}^{2}$ = 0.16). There was a large effect size for HR, with visualization (**Figure 1C_SUP_**) suggesting an increase in the GRP session.

For the comparison of competitive stimulus, the change in TT duration between 1v1 and GRP sessions was not significantly different (*P* = 0.10, *d* = 0.74) between the INF (-5.41 ± 10.82-s) and SUP (2.05 ± 10.22-s) groups. There was, however, a moderate effect size. Overall, nine INF participants (**Figure 2C_INF_**) and 6 SUP participants (**Figure 2C_SUP_**) beat their 1v1 TT duration in the GRP session.

### Analysis of Goal Orientation

**FIGURE 3:**
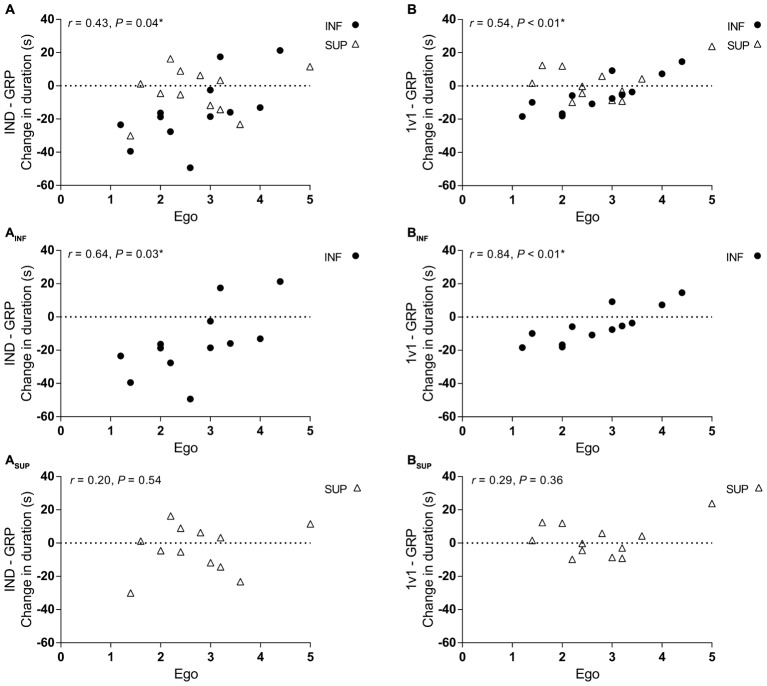
Correlation between ego orientation and change in TT duration between sessions. **(A,A_INF,_A_SUP_)** Correlation between ego orientation and change in TT duration between IND and GRP sessions for all participants (*n* = 24) **(A)**, INF participants (*n* = 12) **(A_INF_)**, and SUP participants (*n* = 12) **(A_SUP_)**. **(B,B_INF,_B_SUP_)** Correlation between ego orientation and change in TT duration between 1v1 and GRP for all participants (*n* = 24) **(B)**, INF participants (*n* = 12) **(B_INF_)**, and SUP participants (*n* = 12) **(B_SUP_)**. IND, individual TT; 1v1, two matched participants TT; GRP, TT with four participants (two INF and two SUP participants); INF, inferior (black circle); SUP, superior (white triangle). Correlation data presented as Pearson correlations (*r*). ^∗^Significant correlation.

There was no significant correlation for change in TT duration between IND and 1v1 for ego (*r* = 0.12, *P* = 0.59) or task (*r*_s_ = 0.09, *P* = 0.66). For all participants, ego orientation displayed significant correlations for a change in TT duration between GRP session and both the IND (**Figure 3A**) and 1v1 (**Figure 3B**) sessions. When analyzed based on groups, ego orientation correlations were significant for changes in TT duration between IND–GRP and 1v1–GRP for INF (**Figures 3A_INF_,B_INF_**) but not the SUP group (**Figures 3A_SUP_,B_SUP_**).

**FIGURE 4:**
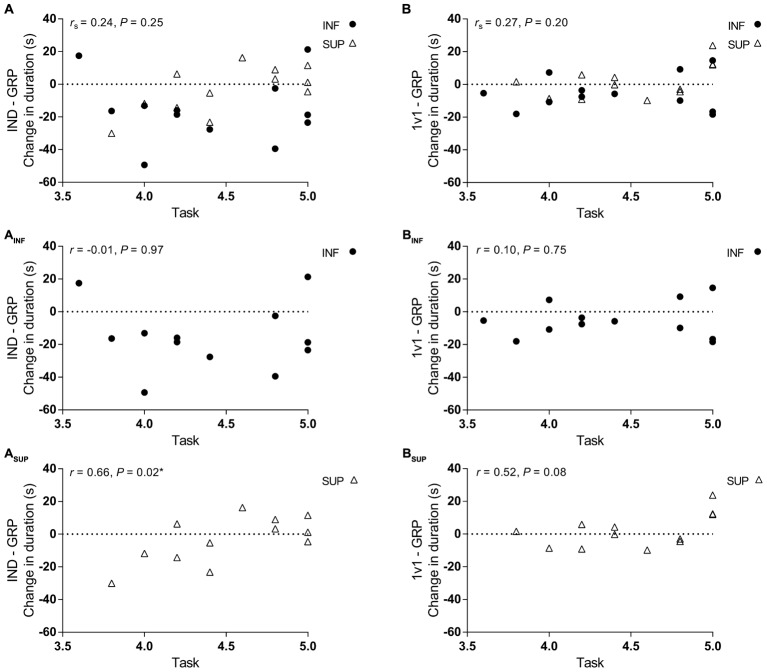
Correlation between task orientation and change in TT duration between sessions. **(A,A_INF,_A_SUP_)** Correlation between task orientation and change in TT duration between IND and GRP sessions for all participants (*n* = 24) **(A)**, INF participants (*n* = 12) **(A_INF_)**, and SUP participants (*n* = 12) **(A_SUP_)**. **(B,B_INF,_B_SUP_)** Correlation between task orientation and change in TT duration between 1v1 and GRP for all participants (*n* = 24) **(B)**, INF participants (*n* = 12) **(B_INF_)**, and SUP participants (*n* = 12) **(B_SUP_)**. IND, individual TT; 1v1, two matched participants TT; GRP, TT with four participants (two INF and two SUP participants;. INF, inferior (black circle); SUP, superior (white triangle). Correlation data presented as Pearson correlations (*r*). When normality of data was violated, correlation is reported as Spearman’s rho (*r_s_*). ^∗^Significant correlation.

For all participants, there was no significant correlation between task orientation and change in performance between any session (**Figures 4A,B**). However, when analyzed based on groups, there was a significant correlation between task orientation and a change in TT duration between IND and GRP for the SUP group (**Figure 4A_SUP_**). Although not significant, there was a large correlation for a similar effect between 1v1 and GRP sessions for the SUP participants (**Figure 4B_SUP_**).

### Analysis of Motivational Orientation and Perceptual Scores

Analysis of motivational responses revealed no significant difference between sessions for interest/enjoyment, perceived competence and pressure/tension. For analysis within the INF and SUP groups, there was no significant difference in any motivational response. However, a moderate effect size for pressure/tension in the INF group (*P* = 0.07, $\eta_{p}^{2}$ = 0.11) indicates an increased pressure/tension for the GRP session (3.75 ± 1.39) compared to the IND (2.80 ± 1.02) and 1v1 (3.10 ± 1.12) sessions. In the GRP session, the perceived competence of the SUP group (5.15 ± 1.38) was significantly greater (*P* = 0.02, *d* = 1.07) than the INF group (3.78 ± 1.30). There was no significant mean change or within group change for RPE or FS at any TT distance, this was also the case for the analysis of the INF and SUP groups.

### Analysis of Pacing Profiles

**FIGURE 5:**
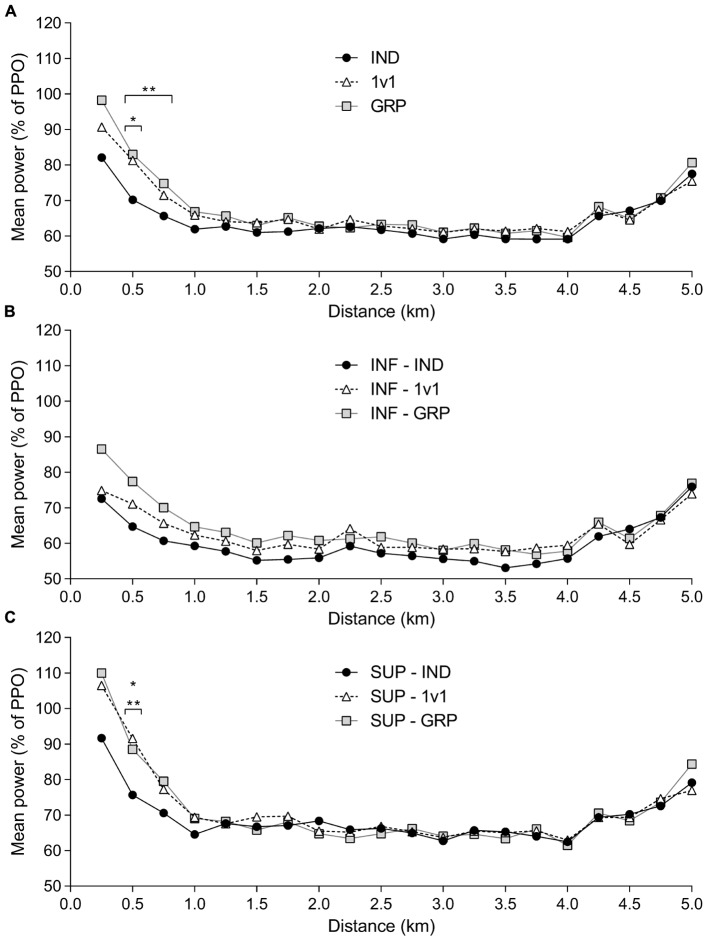
Mean power output pacing profiles. Mean power output averaged over 250-m intervals for all participants (*n* = 24) **(A)** between IND (black circle), 1v1 (white triangle) and GRP (gray square). Mean power output averaged over 250-m intervals for INF participants **(B)**. Mean power output averaged over 250-m intervals for SUP participants **(C)**. INF, inferior; SUP, superior; IND, individual TT; 1v1, two matched participants TT; GRP, TT with four participants (two INF and two SUP participants). ^∗^Significant difference between IND and 1v1. ^∗∗^Significant difference between IND and GRP.

Pacing profiles for all participants are shown in **Figure 5A**. IND power output was significantly lower compared to 1v1 and GRP at 500-m (*P* = 0.01, $\eta_{p}^{2}$ = 0.12), and only significantly lower than GRP at 750-m (*P* = 0.03, $\eta_{p}^{2}$ = 0.09). At 250-m there was no significant difference (*P* = 0.07, $\eta_{p}^{2}$ = 0.07), although there was a moderate effect size.

There was no significant difference between trials for the INF group (**Figure 5B**), although there were moderate effect sizes at 250-m (*P* = 0.12, $\eta_{p}^{2}$ = 0.12), 500-m (*P* = 0.13, $\eta_{p}^{2}$ = 0.12), and 750-m (*P* = 0.16, $\eta_{p}^{2}$ = 0.11). For the SUP group, mean power was significantly lower in IND compared to 1v1 and GRP at 500-m (*P* < 0.01, $\eta_{p}^{2}$ = 0.25) (**Figure 5C**). There was no significant difference, but moderate effect sizes at 250-m (*P* = 0.15, $\eta_{p}^{2}$ = 0.11) and 750-m (*P* = 0.10, $\eta_{p}^{2}$ = 0.13).

Given these changes in pacing profiles, Pearson correlation coefficients (*r*) were calculated for ego and task scores from the GOEM and the change in mean power within the first 750-m for both INF and SUP groups. However, there was no significant correlation for ego or task between any competition setting.

## Discussion

This study was the first to investigate the influence of actual competition with one and multiple competitors, as well as the effect of goal and motivational orientation on cycling TT pacing and performance. No significant change in overall performance was found when competing against one or multiple competitors. Yet, changes in HR and large effects for power output indicate an increased intensity in the GRP competition session. Additionally, in the GRP competition session, significant correlations were found indicating that higher ego orientations result in diminished performance improvements when competing against superior opponents.

### Comparison Between Competition Sessions

The presence of actual competition in the 1v1 and GRP competition settings produced no significant change in 5-km TT performance for all participants. However, a large effect for mean power output for INF participants indicates increased work in the GRP session compared to IND (**Figure 1B_INF_**). In addition to this, HR was greater in GRP compared to IND for all participants (**Figure 1C**), with large effects sizes for both INF (**Figure 1C_INF_**) and SUP (**Figure 1C_SUP_**) participants. To explain this, HR may reflect increased arousal in the presence of others (Bond and Titus, 1983), which is increased further with multiple competitors (Cooke et al., 2013). Taken together with the observed change in power output, these results indicate a slightly increased exercise intensity at the beginning of the TT that can be attributed to the presence of competitors in the GRP session. Given the previous research with the perception of a 1v1 competitor (Corbett et al., 2012) and the presence of competitors in a group setting (Tomazini et al., 2015), these results of an increased intensity were expected compared to exercising alone. Consequently, we aimed to investigate any possible difference in performance between these two differing competition settings.

A direct comparison between the 1v1 and GRP sessions indicates no difference in mean performance measures (**Figures 1A,B**). When investigating individual results, 15 out of 24 participants improved their time in GRP compared to 1v1 (**Figure 2C**). Of these 15 participants, nine were INF participants (**Figure 2C_INF_**) and six were SUP participants (**Figure 2C_SUP_**). Accordingly, in comparison to the 1v1 competition, this suggests our GRP session design provides lower levels of benefit for SUP participants. In fact, performance in both the 1v1 and GRP sessions was identical for SUP participants (**Figures 1A_SUP_,B_SUP_, 5C**). Therefore, it appears the added INF competitors were of no benefit to the SUP participants as they did not provide an interaction that required the SUP participants to respond (Smits et al., 2014). Indeed, this finding is consistent with social facilitation theory, in that performance is likely to be affected more by the presence of others if they are perceived to be important competitors (Bond and Titus, 1983). Accordingly, for the INF participants, the addition of SUP competitors provides a motivational stimulus greater than the 1v1 session, resulting in enhanced performance, explained by the large effect on mean power (**Figures 1A_INF_,B_INF_**). Consequently, this demonstrates that the presence of a competitor must be an appropriate motivational stimulus for any potential performance improvement (Bond and Titus, 1983; Bath et al., 2012).

In terms of pacing profiles, the presence of the SUP competitors in the GRP session was associated with a large effect on power output in the first 750 m of the TT for the INF participants (**Figure 5B**). Explaining this, the addition of the SUP competitors creates a stimulus for the INF participants to change their pre-established pacing profile. Consequently, the motivation to be competitive results in an increased power output to match the SUP opponents (Konings et al., 2016). However, matching the superior opponents is more physically demanding (Lander et al., 2009) and this would create a greater metabolic disturbance requiring management of pace in order to avoid detrimental metabolic consequences (St Clair Gibson et al., 2006). At the start of the TT, this afferent information is not accurately considered as the attentional focus is shifted away from internal aspects relating to the physiological status and toward the behavior of competitors (Williams et al., 2015a). However, as afferent information becomes more prominent, regulation will be necessary, and accordingly, after 750-m, GRP pacing follows a similar profile to IND and 1v1 for the INF participants. For the SUP participants, power output was similarly increased in both 1v1 and GRP compared to IND in the first 750-m (**Figure 5C**). In addition to our mean performance results, this indicates our GRP competition provides no additional benefit or change in exercise strategies. These results are likely due to the behavior of the nearest competitor being unchanged between conditions, with the added presence of the INF participants not influencing SUP performance.

Although the INF participants increased power output at the start of the GRP TT (**Figure 5B**), power output was still relatively greater for the SUP participants (**Figure 5C**) during this part of the TT. Consequently, the difference between the INF and SUP participants would be well established in the initial stages of the GRP TT. This is an important factor to consider for group exercise settings, as a negative perception of competence can decrease motivation (Ryan and Deci, 2017) and result in performance reductions (Mauger et al., 2011). In fact, post TT ratings demonstrate the difference in perceived competence between the participant groups. Additionally, a moderate effect size indicates a likely increase in pressure/tension for INF in the GRP TT. Taking these results into account, it would be expected that INF participants would have reduced performance in the GRP session. Yet the large effect of mean power and no change in interest/enjoyment indicates this was not the case (**Figure 1B_INF_**). Although the INF participants perceived themselves as less competent than their SUP counterparts, it is likely motivation was still adequate from their INF 1v1 opponent. However, another possible explanation for these changes in performance is the way individuals approach a task based on goal orientations.

### Influence of Goal Orientation

In conjunction with investigating the possible differences between competitive settings, the secondary aim of our study was to investigate the impact of goal orientations on performance within our competitive environments. It was hypothesized that based on personal goals, the interaction of multiple competitors may result in responders and non-responders to the differing exercise conditions. In fact, within the GRP session, there was a significant relationship between ego orientation of the INF participants competing against SUP competitors (**Figures 3A_INF_,B_INF_**), while no correlation for the SUP participants competing against INF participants was found (**Figures 3A_SUP_,B_SUP_**). These results demonstrate that when competing against SUP opponents, ego-oriented individuals are less likely to respond to the presence of a SUP competitor and improve performance. This is due to ego individuals evaluating performance on social comparison and perceptions of competence (Nicholls, 1984). With a difference in the competence perceptions between our groups in the GRP session, ego orientated INF individuals likely exhibited negative achievement behaviors allowing them to avoid disgrace by not achieving their goal through lack of effort (Nicholls, 1984). This appears to be the case as the highest ego orientated INF participants accounted for diminished performance in the GRP session (**Figures 3A_INF_,B_INF_**).

Another explanation of these results is the instruction given to participants that created a more task involving scenario. For all competition TTs, participants were instructed to do their best while they ride with individuals which may be slower or faster than them. This means that, even for those who exhibit high ego orientations, the goal of each TT is self-improvement, which presumably diminished the importance of ego goals and appealed to the participant’s goals of task mastery and improvement (Reinboth and Duda, 2016). In fact, as evidence of this, there was no significant end spurt in the 1v1 or GRP sessions as participants did not increase power output to beat a competitor, although this has been demonstrated previously with competition (Corbett et al., 2012; Stone et al., 2012). Given the task involving scenario, it is surprising that there was a correlation for the SUP participants in the GRP TT (**Figure 4A_SUP_**), with those with greater task orientations having reductions in performance. It is unclear as to why this is the case, it may be that these participants are conflicted by the presence of the competitors. Along with this line of reasoning, the presence of competitors provides external sensory input and reduces internal attentional focus (Williams et al., 2015a) which may be a conflict for task individuals that does not enable them to focus on their goal of the exercise. Nevertheless, this result is another indication that the addition of INF participants in a GRP TT, is of no additional benefit to SUP participants.

### Limitations

Given the design of our research and instructions to participants, we have inadvertently created a task involving a scenario that may limit our conclusions as to how ego and task individuals respond to differing competition settings. It was anticipated that if participants were free to dictate outcome (i.e., employing tactics) that this would be a detriment to performance (Thiel et al., 2012). Fundamentally, tactics will likely hinder the best performance but increase the likelihood of a positive competitive outcome (Hettinga et al., 2017). Therefore, for this investigation, we looked at the improvement that competition may provide when the effort is maximal, but the investigation of tactical components and goal orientations may be an area for future research. Additionally, in highlighting the difference between actual and virtual competition, within this study we have only utilized actual competition, and have not addressed a direct comparison between real competition and an avatar for this mode of exercise. Ultimately, the use of actual competition represents a strength of this study as it improves ecological validity. Yet, the use of actual competition also provides several limiting variables compared to an avatar, including the inability to standardize the 1v1 and GRP competitors, as well as variability in matching participants and controlling for differences in ability.

## Conclusion

The presence of a competitor is known to influence pacing and performance. However, this study found no significant difference in 5-km TT performance between 1v1 or GRP competition settings. Yet, large effects on power indicate that INF participants are motivated to match SUP competitors in the initial stages of GRP exercise that may lead to small improvements in overall performance. Yet in a GRP setting, SUP participants may be detrimental to INF participants who are ego orientated, while INF participants provide no benefit to the performance of SUP participants. Overall, these findings demonstrate that competition is an important determinant of pacing and performance, and consideration should be given to the ability of competitors in a group setting to provide adequate motivation to achieve performance improvements.

## Author Contributions

AH, FB, MV, and RP: conceived and designed the experiments, edited and critically revised the manuscript, and approved the final version of manuscript. AH and RP: interpreted results of research. AH: analyzed the data, drafted the manuscript, and prepared the table and figures.

## Conflict of Interest Statement

The authors declare that the research was conducted in the absence of any commercial or financial relationships that could be construed as a potential conflict of interest. The reviewer LV and handling Editor declared their shared affiliation.
